# Dynamic Regulation of the Nexus Between Stress Granules, Roquin, and Regnase-1 Underlies the Molecular Pathogenesis of Warfare Vesicants

**DOI:** 10.3389/fimmu.2021.809365

**Published:** 2022-01-10

**Authors:** Ritesh Kumar Srivastava, Bharat Mishra, Suhail Muzaffar, Marina S. Gorbatyuk, Anupam Agarwal, M. Shahid Mukhtar, Mohammad Athar

**Affiliations:** ^1^ University of Alabama at Birmingham (UAB) Research Center of Excellence in Arsenicals, Department of Dermatology, University of Alabama at Birmingham, Birmingham, AL, United States; ^2^ Department of Biology, University of Alabama at Birmingham, Birmingham, AL, United States; ^3^ Department of Optometry and Vision Science, School of Optometry, University of Alabama at Birmingham, Birmingham, AL, United States; ^4^ Department of Medicine, University of Alabama at Birmingham, Birmingham, AL, United States; ^5^ Department of Veterans Affairs, Birmingham Veterans Administration Medical Center, Birmingham, AL, United States

**Keywords:** arsenicals, inflammation, RNA-binding proteins, stress granules, skin

## Abstract

The use of chemical warfare agents is prohibited but they have been used in recent Middle Eastern conflicts. Their accidental exposure (e.g. arsenical lewisite) is also known and causes extensive painful cutaneous injury. However, their molecular pathogenesis is not understood. Here, we demonstrate that a nexus of stress granules (SGs), integrated stress, and RNA binding proteins (RBPs) Roquin and Reganse-1 play a key role. Lewisite and its prototype phenylarsine oxide (PAO) induce SG assembly in skin keratinocytes soon after exposure, which associate with various RBPs and translation-related proteins. SG disassembly was detected several hours after exposure. The dynamics of SG assembly-disassembly associates with the chemical insult and cell damage. Enhanced Roquin and Regnase-1 expression occurs when Roquin was recruited to SGs and Regnase-1 to the ribosome while in the disassembling SGs their expression is decreased with consequent induction of inflammatory mediators. SG-targeted protein translational control is regulated by the phosphorylation-dependent activation of eukaryotic initiation factors 2α (eIF2α). Treatment with integrated stress response inhibitor (ISRIB), which blocks eIF2α phosphorylation, impacted SG assembly dynamics. Topical application of ISRIB attenuated the inflammation and tissue disruption in PAO-challenged mice. Thus, the dynamic regulation of these pathways provides underpinning to cutaneous injury and identify translational therapeutic approach for these and similar debilitating chemicals.

## Introduction

Exposure to arsenical vesicants rapidly causes severe and painful inflammation and blistering responses, primarily in the skin but also in other epithelial tissues. In light of these effects, arsenical vesicants were developed during World War I and II as chemical weapons capable of debilitating military and civilian populations (1, 2). Lewisite, was considered to be one of the most toxic and leading candidates for weaponizing arsenicals, as a single agent and as a complex mixture with sulfur mustard, another potent vesicant, for use in World War II (3). Fortunately, these chemicals were not used due to war accords, and stockpiles were eventually buried at the manufacturing site or sunk in the ocean (4). Despite the efforts of the Organization for the Prohibition of Chemical Weapons, which oversees the timely destruction of these agents and works to ensure that these chemicals are not synthesized and stockpiled, large stockpiles of these and similar chemicals still exist in numerous countries (5). These stockpiles, along with the ease of synthesizing these agents, may enable their use by terrorist groups. According to Human Rights Watch, 85 chemical weapon attacks by the Syrian government or by terrorist organizations were noted between August 2013 and February 2018 (6). Some of these attacks included the use of vesicants. Furthermore, buried or ocean-dumped arsenicals pose a significant threat to human and environmental health due to the accidental exposure or decay of shells containing these toxic agents. For example, diphenylarsinic acid, a degradation product of diphenylchlorarsine or diphenylcyanoarsine, also known as Clark-I and Clark-II, was found in drinking water in Kamisu, Japan. Consumption of this contaminated water caused neuronal syndrome with cerebellar symptoms among Kamisu residents (7). In many European and Asian countries, buried ammunition including arsenicals from World War II found during excavations posed threats to the residents of these areas (8). For instance, 44 victims were poisoned by the mixture of sulfur mustard and lewisite leaking from five drums excavated from an underground parking area in Qiqihar City of Heilongjiang Province, China. One of these victims died 17 days after exposure. The remaining victims survived but developed long-term neurological and neuropsychological complications (9). Similarly, in Samukawa, Kanagawa, where the Sagami Naval Arsenal was formerly located, many workers were accidently exposed to lewisite (10).

Effective treatment options for preventing vesicants including arsenical-induced injury are lacking, however, because the molecular mechanisms involved in the pathobiologic response have not been well characterized. Studies of these molecular mechanisms have been hindered by the lack of a relevant animal model and the need to conduct these studies in specialized laboratories designed for the study of highly toxic agents. To overcome these obstacles, we recently developed a sensitive murine model, Ptch1^+/-^/SKH-1, with which the molecular pathogenesis of lewisite can be investigated (2, 11). We also developed a surrogate arsenical, phenylarsine oxide (PAO), which is less toxic than lewisite and can be used in the standard research laboratory with proper use of personal protective equipment (12). We demonstrated that cutaneous exposure to PAO causes microscopic histopathological changes similar to those caused by lewisite. Our recent studies with these tools revealed that the pathogenic effects of cutaneous exposure to lewisite and PAO are mediated by the induction of endoplasmic reticulum (ER) stress, which leads to the accumulation of unfolded proteins and activation of the unfolded protein response (UPR) signaling pathway (12). However, the additional molecular mechanisms involved in the injury resulting from cutaneous exposure to arsenicals remain unclear.

In response to acute ER stress, cells often form stress granules (SGs) in addition to activating UPR signaling (13). These cytoplasmic multi-molecular aggregates of stalled translation pre-initiation complexes entrap untranslated mRNAs and mRNA-associated proteins, further inhibiting global protein synthesis and the accumulation of misfolded proteins (14, 15). Classically, the stress-induced assembly of SGs involves the aggregation of specific RNA-binding proteins (RBPs), including poly (A) binding protein (PABP), Hu antigen R (HuR), T-cell intracellular antigen-1 (TIA1), and Ras-GAP SH3 domain binding protein (G3BP1) (15–18), and activated translation initiation factors such as eIF3A, eIF4G, and eIF2α (19, 20). Although SG formation has been shown to help protect cells from a variety of toxic insults (21), the potential role of SGs in the pathogenic response to arsenicals has not been evaluated.

Furthermore, we uncover the role of SGs in arsenical-induced cutaneous inflammation and tissue injury. We characterize the dynamics of SG assembly and disassembly and show that this process coincides closely with lewisite or PAO-mediated skin injury. Subsequently, we demonstrate a critical role of the RBPs Roquin (also known as RC3H1) and Regnase-1 in arsenical-mediated skin damage. Mechanistically, arsenical-induced phosphorylation of eIF2α is involved in the formation of SGs and inhibiting phosphorylation of eIF2α with integrated stress response inhibitor (ISRIB), a potent inhibitor of eIF2α, retards the arsenical-induced formation of SGs as well as toxicity. Administration of ISRIB after PAO exposure afforded significant protection against skin inflammation and associated injury. These protective effects were linked to restoration of the expression of Roquin and Regnase-1 in the arsenical-challenged skin.

In this manuscript, we document the role of SGs in arsenical-induced cutaneous inflammation and tissue injury, as well as the temporal alterations in skin defense mechanisms in response to arsenical exposure, providing a critical step in understanding the molecular basis of hazardous nature of these and similar chemicals. Furthermore, we uncover the mechanisms underlying the robust inflammatory signaling associated with arsenical-induced cutaneous blistering. Importantly, we also identify a highly effective small molecule that may be developed into a novel mechanism-based antidote for arsenicals.

## Materials and Methods

### Cell Lines and Reagents

Human skin keratinocytes (HaCaT), purchased from AddexBio Technologies (San Diego, CA, USA), were cultured in DMEM medium (Hyclone, South Logan, UT, USA) containing 10% fetal bovine serum (FBS; Sigma, St. Louis, MO, USA) and 1% antibiotic solution (Mediatech, Manassas, VA, USA). PAO was obtained from Sigma. ISRIB was procured from Apex BIO (Houston, TX, USA). siRNA against Roquin (Cat No. SI00462224) and Regnase-1 (Cat. no. SI03144470) were obtained from Qiagen (Hilden, Germany). The Mouse Cytokine Antibody Array C 3 kit was obtained from Ray Biotech (Norcross, GA, USA). RT- PCR primers used in the study were obtained from ThermoFisher Scientific (Grand Island, NY, USA) and are provided in **Supplementary Table-S1**. The description of antibodies used in this study is provided in **Supplementary Table-S2**.

### Cell Culture Treatments

PAO (1M stock) was prepared fresh in 100% DMSO by warming at 37°C for 5–10 min as described previously (11). HaCaT cells were treated either with vehicle control or with various concentrations (0.062–1.0 µM) of PAO diluted in culture medium for up to 12 h. To assess protection against PAO-induced effects, cells were co-treated with PAO and ISRIB (100 nM). All *in vitro* studies were performed in cultures that were 60-70% confluent.

### Animal Studies

Ptch1^+/-^/SKH-1 mice (2, 12) were used in this study. Both male and female mice aged between 10-12 weeks were used. However, these studies were not planned to identify the impact of gender on the experimental-results as additional animals should be incorporated to clearly define the sex dependent responses. Future studies will clarify if sex differences exist in this model. Prior to cutaneous lewisite or PAO challenge, mice were anesthetized with ketamine (100 mg/kg) and xylazine (5 mg/kg) by intraperitoneal injection. To manage pain, buprenorphine (0.05-0.10 mg/kg) was administered 30 min prior to anesthesia. The anesthetized animals were topically treated with either ethanol-diluted lewisite (94 μL applied on the dorsal skin over 8 cm^2^ at a dose of 5 mg/kg) or ethanol-diluted PAO (30 μL applied on the dorsal skin over 2×2 cm^2^ at a dose of 100 µg/mouse). Control mice were similarly treated with vehicle (ethanol) alone. Lewisite exposure experiments were performed at MRIGlobal (Kansas City, MO, USA) as described previously (2). However, PAO exposure experiments were performed in our laboratory in the Department of Dermatology, University of Alabama at Birmingham. All the animal protocols were approved by the Institutional Animal Care and Use Committee of the University of Alabama at Birmingham and MRIGlobal. Dose selection for lewisite and PAO was based on our previously published studies (2, 12). Lewisite-treated animals were euthanized 6–72 h after treatment and tissues were excised. In efficacy studies, ISRIB (200 µg/mouse) was applied topically to mice 5–10 min after PAO exposure. Efficacy studies were terminated at 6 h following PAO exposure, as our *in vitro* studies showed that blocking the production of SGs early after cutaneous arsenical exposure could diminish inflammatory signaling and further tissue injury.

### Histology and Immunohistochemical Examination

Hematoxylin and eosin (H&E) staining was performed as described previously (11). Briefly, skin tissue was fixed in 10% buffered formalin and embedded in paraffin, then cut into 5-μm sections using a microtome (HM 325, ThermoFisher, Grand Island, NY, USA). At least 3 independent skin tissue sections from each group were H&E stained and examined for histological changes using a Keyence Fluorescence Microscope Model BZ-X710 (Osaka, Japan).

Immunohistochemistry (IHC) was conducted with a rabbit-specific IHC polymer detection kit (Abcam, Cambridge, MA, USA) per the manufacturer’s protocol. The macrophage marker F4/80 was detected using antibody (Cat No. ab100790, Abcam) with 3,3’-diaminobenzidine substrate (Abcam), and slides were examined under the microscope.

### Immunofluorescence Staining

For immunofluorescence staining, skin sections were deparaffinized, rehydrated, and then incubated in antigen unmasking solution following the manufacturer’s instructions (Vector Laboratories, Burlingame, CA, USA). Sections were then incubated in a blocking buffer of 2% bovine serum albumin in PBS for 30 min at 37°C to avoid non-specific binding of antibodies and incubated with primary antibodies overnight at 4^0^C. The next day, sections were incubated with fluorescence-coupled secondary antibody and visualized with a Keyence Fluorescence Microscope Model BZ-X800. Immunofluorescence staining of cultured keratinocytes was performed on cells fixed with 4% paraformaldehyde and permeabilized with 0.5% Triton X-100 as described previously (22).

### Comparative Omics Analyses

The comparative transcriptomic analysis of SG-associated protein expression (23) was performed on publicly available RNA binding proteomes in three cell lines representing the translational arrest (24) with an in-house 24-h lewisite-exposed skin transcriptome (total RNAseq). The threshold for significant differential expression was |1| for log_2_ fold change and a false discovery rate-adjusted p value <0.05 was considered significant. Pathway enrichment analysis was performed with the Ingenuity Pathway Analysis platform with significance set at p<0.05.

### Gene Expression Analysis Using nanoString

RNA (100 ng) from vehicle or lewisite-exposed skin samples was hybridized overnight at 65°C to the nanoString nCounter mouse Inflammation V2 panel (nanoString Technologies, Seattle, WA, USA) comprising 254 genes. Hybridized samples were then immobilized onto an nCounter cartridge and imaged on an nCounter SPRINT Profiler (nanoString Technologies) as previously described (25). Data were analyzed with nSolver Analysis Software (nanoString Technologies). The threshold for significant differential expression was |1| for log fold change and p<0.05 for t-test significance.

### Cytokine Antibody Arrays

Precleared protein (50 µg) lysates of murine skin from at least 5 individual mice from each group were pooled and incubated with a RayBio Mouse Cytokine Antibody Array C3 membrane (CODE: AAM-CYT-3-2) according to the manufacturer’s protocol (RayBiotech, Norcross, GA, USA). The Mouse Cytokine Antibody Array C3 membrane was used to evaluate the pattern of expression for 62 murine cytokines, following instructions in the user manual. X-ray films were analyzed by Excel-based analysis software tools (RayBiotech) for the automatic computation of the extracted numerical data obtained from the array images.

### Real-time PCR Analysis

Real-time PCR (RT-PCR) analysis was performed using TaqMan One-Step PCR Master Mix (Applied Biosystems, Foster City, CA, USA) as described previously (12). Total RNA was extracted from either HaCaT cells or skin tissues using 1 mL TRIzol reagent (Invitrogen, Grand Island, NY, USA). The isolated total RNA (1 μg) was reverse transcribed into cDNA with an iScript cDNA synthesis kit (Bio-Rad, Hercules, CA, USA). qPCR reactions were carried out with a 7500 Fast RT- PCR System (Applied Biosystems) using Taqman primers as described in **Supplementary Table-S1**. Relative quantification of the mRNA levels was calculated after the total amount of cDNA was normalized to GAPDH as an endogenous control; the fold change in gene expression is presented.

### TUNEL Assay

Terminal deoxynucleotidyl transferase dUTP nick-end labeling (TUNEL) assays were performed using a commercial apoptosis detection kit (Roche Diagnostics, Indianapolis, IN, USA) following the manufacturer’s instructions. The TUNEL-stained slide sections were mounted with anti-fade DAPI and visualized with a Keyence Fluorescence Microscope Model BZ-X710.

### Protein Quantification and Western Blot Analysis

Protein lysates from HaCaT cells and skin tissues were collected in ice-cold lysis buffer (Bio-Rad). Protein concentrations in cell lysates were determined using a DC kit (Bio-Rad). These samples were separated by SDS-PAGE electrophoresis and transferred onto a PVDF membrane. Following protein transfer, nonspecific sites were blocked with 5% nonfat dry milk in Tris buffered saline plus Tween-20 for 1 h at room temperature. Membranes were then incubated with primary antibodies overnight at 4°C. The next day, blots were washed three times for 10 min each on an orbital shaker, then membranes were incubated with HRP-conjugated secondary antibodies for 1.5 -2.0 h. Protein bands were visualized with an iBright1000 imaging system with the help of enhanced chemiluminescence according to the manufacturer’s instructions (Santa Cruz Biotechnology, Dallas, TX, USA).

### siRNA Transfection

For siRNA-mediated knockdown studies, human keratinocytes were transfected with siRNA against human Roquin or Regnase-1 or with scrambled siRNA (negative control), at a final concentration of 5 nM using Lipofectamine 2000 (Invitrogen, Carlsbad, CA, USA) according to the manufacturer’s instructions. Knockdown efficiency was confirmed by RT-PCR and Western blot analysis.

### Statistical Analysis

GraphPad Prism was used for statistical analysis. Data are reported as the mean ± standard error of the mean (SEM). At least p<0.05 was reported as a significant change. Student’s t-test was done for analysis between two groups, however, multiple treatment groups were analyzed using either one-way or two-way analysis of variance (ANOVA).

## Results

### Induction of SGs in Arsenical-Treated Human Skin Keratinocytes and Murine Skin

**Figure 1:**
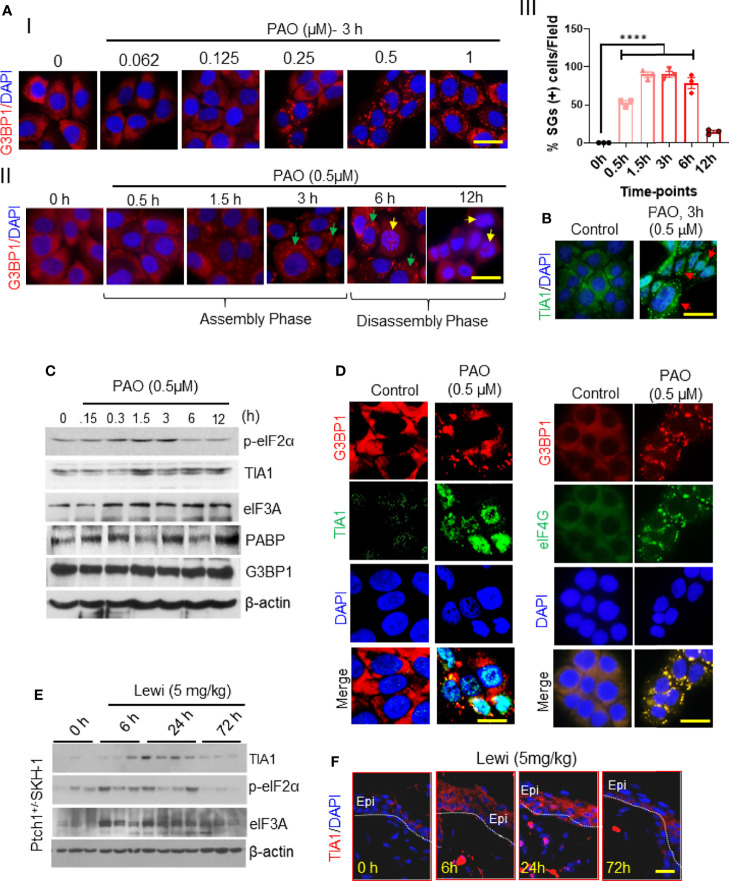
Arsenical-mediated induction of SGs in human skin keratinocytes and murine skin. **(AI-III)** G3BP1 expression was detected in HaCaT cells before and after PAO exposure. **(I)** Representative immunofluorescence imaging of the dose-dependent effects of PAO (0.062-1 µM) on G3BP1-positive SG assembly (red) 3 h after exposure. Nuclei were stained with DAPI (scale bar 50 µm). **(II)** Representative immunofluorescence imaging of the time-dependent effects of PAO (0.5 µM) on G3BP1-positive SG (red) assembly and disassembly. Green arrows point to G3BP1-positive SGs; yellow arrows point to cells in which G3BP1 has returned to a diffuse staining pattern, indicative of SG disassembly, and apoptotic nuclear body formation is apparent. Nuclei were stained with DAPI (scale bar, 50 µm). **(III)** Quantitative analysis of the time-dependent changes in the percentage of SG-positive cells detected after PAO (0.5 µM) exposure. **(B)** Representative immunofluorescence imaging of TIA1 expression (green) in HaCaT cells detected 3 h after PAO (0.5 µM) exposure. Nuclei were stained with DAPI. **(C)** Representative Western blots showing the time-dependent effects of PAO (0.5 µM) exposure on the expression of p-eIF2α, TIA1, eIF3A, PABP, and G3BP1 in HaCaT cells. β-actin was used as the endogenous loading control. **(D)** Representative immunofluorescence imaging of G3BP1 (red) and TIA1 (green; left panels) or eIF4G (green; right panels) expression in HaCaT cells 3 h after PAO (0.5 µM) exposure. Nuclei were stained with DAPI (scale bar 50 µm). **(E)** Representative Western blots showing the time-dependent effects of cutaneous lewisite (5 mg/kg) exposure on the expression of p-eIF2α, TIA1, and eIF3A in the skin of Ptch1^+/-^/SKH-1 mice. β-actin was used as the endogenous loading control. **(F)** Representative immunofluorescence imaging of the time-dependent changes in TIA1 expression (red) in skin sections from Ptch1^+/-^/SKH-1 mice cutaneously exposed to lewisite (5 mg/kg). Nuclei were stained with DAPI. (scale bar 50µm). Lewi, lewisite; Epi, epidermal cells. p < 0.0001**** show significant.

To begin probing the effect of arsenical exposure on SG assembly in skin cells, we treated cultured immortalized human keratinocytes (HaCaT cells) with PAO. Immunofluorescence staining of cultured keratinocytes to detect G3BP1 revealed that PAO induced SG assembly in a dose- and time-dependent manner (**Figure 1**), with 0.5 µM PAO sufficient to induce SG assembly in more than 95% of the cells (**Figures 1AI–III**). The assembly of G3BP1-containing SGs was evident by 0.5 h and peaked by 3 h after exposure (**Figures 1AI, II**). This assembly phase could provide an important protective mechanism in cells before the toxic effects of these chemicals are manifested. It is also known that the contents of SGs depend on the nature of toxic agents to which cells are exposed as well as on the cell context (23). However, in this study, we linked the dynamics of assembly and disassembly of SGs to the pathobiology of arsenicals in skin keratinocytes and in the skin. By 6 h after PAO exposure, however, diffuse localization of G3BP1 in the cytoplasm was apparent, and by 12 h after exposure, G3BP1-containing SGs were no longer detected. During this 6- to 12-h SG disassembly phase, common indicators of cell apoptosis were detected, including increased expression of cleaved caspase-3 (data not shown) and nuclear apoptotic body formation (**Figure 1AII**).

SG assembly results in rapid repression of general protein translation initiation mediated by the phosphorylation of eIF2α (20). To determine whether this occurs after PAO treatment in keratinocytes as well, we evaluated eIF2α phosphorylation. Western blot analysis demonstrated that after PAO exposure, the level of phosphorylated eIF2α (p-eIF2α) increased over the first 3 h (the SG assembly phase) as did the expression of TIA1 (**Figures 1B, C**). However, during the SG disassembly phase (from 6 to 12 h after exposure), the expression of these proteins and the level of p-eIF2α gradually diminished, although eIF3A and PABP could still be detected 12 h after treatment (**Figure 1C**), and the level of p-eIF2α decreased substantially (**Figure 1C**). However, Western blot analysis did not indicate any change in the protein expression of G3BP1 at any time after PAO exposure (**Figure 1C**). PAO exposure also induced the co-localization of TIA1 and eIF4G with G3BP1 in SGs (**Figure 1D**).

Similar results were observed in murine skin excised from Ptch1^+/-^/SKH-1 mice topically exposed to lewisite (5 mg/kg). The kinetics of SG induction *in vivo* appeared to be quite different from the kinetics in cultured keratinocytes. Western blot analysis showed increased levels of TIA1, p-eIF2α, and eIF3A 6 h and 24 h after lewisite exposure, but this increase was almost completely attenuated 72 h after exposure (**Figure 1E**). Immunofluorescence staining of murine skin samples similarly showed that the expression of TIA1 was substantially higher 6 h and 24 h after lewisite exposure but returned to baseline levels by 72 h (**Figure 1F**). A similar pattern of TIA1 expression was detected in the skin of PAO-treated mice (data not shown). Thus, the time-course of arsenicals-induced SG assembly and disassembly in skin cells differs *in vitro* and *in vivo*, but the pattern is similar, and the *in vitro* studies confirm that this process correlates with the manifestation of cell toxicity.

### Comparative Omics Identified SG Proteins Trigger Translational Arrest During Lewisite-Induced Injury

**Figure 2:**
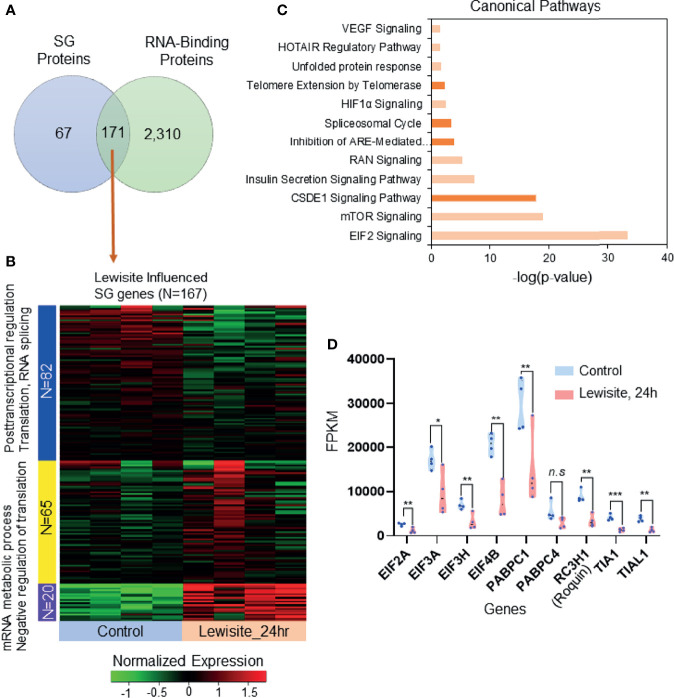
SG-associated proteins trigger translational arrest following cutaneous lewisite exposure. **(A)** A total of 171 proteins are shared between SGs and RBP translational arrest proteomes (also see **Supplementary Figures S1A, B**). **(B)** The transcriptomic profile of the 167 SG RBP proteins in normal and 24 h of lewisite exposure. mRNA expression was normalized and labeled accordingly. Three gene clusters were identified by the k-mean clustering algorithm. **(C)** Significantly enriched canonical pathways identified by Ingenuity Pathway Analysis. **(D)** mRNA expression values (FPKM) of some genes previously reported to be involved in the translational arrest mediated by SGs. p < 0.05*, p < 0.01**, p < 0.001*** show significant and ns- Non significant.

To identify the expression of likely candidate proteins associated with SGs following cutaneous lewisite exposure, we first explored the publicly available data for SGs in three different cell lines, namely MCF7, HeLa, and HEK293 cells (24). Comparative proteomics analysis identified 171 SG-associated proteins that are significantly influenced by the RBP proteome (**Figure 2A**). Most of these proteins are involved in RNA transport, protein processing in ER, RNA degradation, the mRNA surveillance pathway, and the spliceosome, representing the translational arrest during stress, as shown in **Supplementary Figures S1A, B**. Further, we matched the transcriptomic profile of these 171 SG-associated proteins with transcriptomic analysis of skin samples from Ptch1^+/-^/SKH-1 mice 24 h after lewisite exposure. Interestingly, we found that 167 of the 171 SG-associated genes are altered by lewisite exposure at this time-point (**Figure 2B**), which strongly suggest the involvement of SGs in the pathogenesis of skin lesions caused by lewisite. Most of these proteins have been shown to be involved in post-transcriptional regulation, translation, RNA splicing, mRNA metabolic processes, and negative regulation of translation (**Figure 2B**). Ingenuity Pathway Analysis (IPA) of SG associated genes revealed that the canonical pathways in SGs most enriched by lewisite exposure are EIF2 signaling, mTOR signaling, CSDE1 signaling pathway, insulin secretion signaling pathway, RAN signaling, inhibition of ARE-mediated mRNA degradation pathway, spliceosomal cycle, HIF1α signaling, telomere extension by telomerase, unfolded protein response, HOTAIR regulatory pathway, and VEGF signaling pathways (**Figure 2C**; p value<0.05). Consistent with these data, using a luciferase reporter array, we previously identified transcription factors related to ER stress, immune response, and apoptosis signaling pathways that were significantly enhanced in keratinocytes following PAO treatment (2). To further confirm these findings, we extracted the fragments per kilobase million (FPKM) values of some RNA-binding genes in murine skin samples 24 h after lewisite injury. Some of the genes related to translation initiation factors and RBPs, including *Eif2a, Eif3a, Eif3h, Eif4b, Pabpc1, Rc3h1* (Roquin), *Tia1*, and *Tial1*, were significantly downregulated by lewisite (**Figure 2D**).

### Kinetics of the Inflammatory Response Induced by Lewisite or PAO Coincides With the Kinetics of SG Assembly and Disassembly

**Figure 3:**
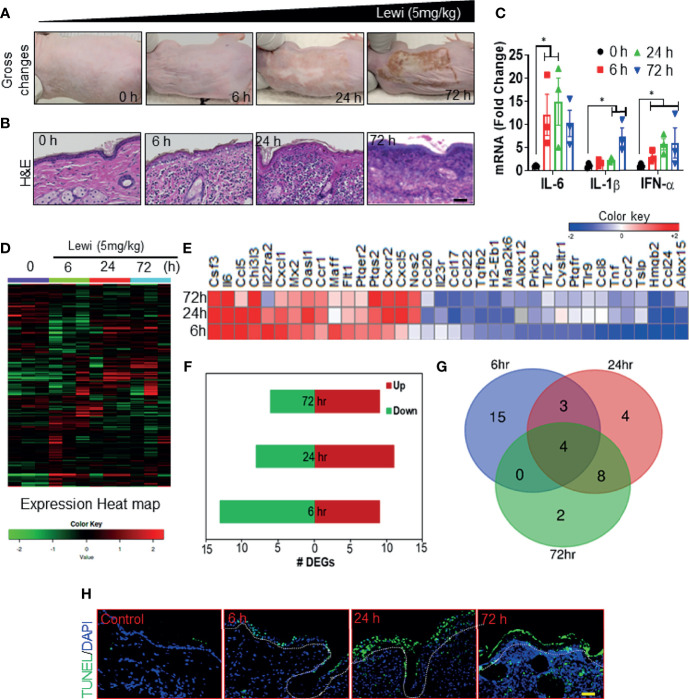
Arsenical-mediated inflammatory and cell death responses coincide with SG assembly and disassembly. Ptch1^+/-^/SKH-1 mice were cutaneously exposed to lewisite (5 mg/kg) for various times. **(A)** Representative images showing the time-dependent effects of cutaneous lewisite (5 mg/kg) exposure on the skin. Gross changes included erythema and wrinkled and leathery skin detected 24 h after exposure and scab formation detected 72 h after exposure. **(B)** Representative H&E-stained skin sections showing the time-dependent histologic effects of cutaneous lewisite (5 mg/kg) exposure. Infiltration of inflammatory cells was obvious at 6 and 24 h after exposure, while necrosis was more prominent 72 h after exposure. (scale bar 50µm). **(C)** Quantitative analysis of the time-dependent effects of cutaneous lewisite exposure on the mRNA expression of inflammatory cytokines, assessed by RT-PCR, in skin. p<0.05* show significance. **(D)** Heatmap showing the time-dependent effects of lewisite exposure on the relative expression of 254 genes in the skin. mRNA expression was normalized and labeled accordingly. **(E)** Heatmap showing the time-dependent effects of lewisite exposure on the relative expression of 36 cumulative differentially expressed genes in the skin. **(F)** Time-dependent effects of lewisite exposure on the number of upregulated and downregulated genes detected in the skin. **(G)** Shared and unique differentially expressed genes identified in the skin of mice at various times after cutaneous lewisite exposure. **(H)** Representative immunofluorescence imaging of the time-dependent effects of lewisite exposure on the detection of TUNEL-positive cells in the skin. Nuclei were stained with DAPI (scale bar 50 µm).

We and others have shown that robust inflammatory and cell death signaling responses accompany the tissue disruption induced by arsenicals (2, 26–28). Here, we examined the correlation between the SG assembly and disassembly phases and the onset of inflammatory and tissue-disrupting responses after arsenical exposure. We hypothesized that early entrapment of pro-inflammatory mRNAs, other mRNAs, and proteins associated with the SG assembly phase would prevent the onset of inflammatory responses largely by blocking protein translation and by inducing the degradation of mRNAs related to regulation of inflammatory and tissue disruption. Conversely, the subsequent disassembly of SGs would facilitate the translation of released pro-inflammatory and apoptosis-inducing mRNAs and thereby initiate tissue damage. Consistent with this notion, we found that in Ptch1^+/-^/SKH-1 mice, cutaneous exposure to lewisite induced a mild injury response in the first 6 h, but the response became more profound and devastating thereafter. By 24 h, the skin became grayish white and leathery, and by 72 h the skin began to slough and deep skin wounds appeared (**Figure 3A**). Histopathologic analysis of H & E-stained tissue sections further revealed that by 72 h, more than 90% of the lewisite-exposed skin showed signs of necrosis in focal areas (**Figure 3B**). Similarly, RT-PCR showed upregulation of the pro-inflammatory cytokines *Il6*, *Il1β*, and *Ifnα* in lewisite-treated skin, and the upregulation of these cytokines differentially coincided with SG assembly or disassembly (**Figure 3C**). Furthermore, nanoString analysis of an inflammation panel of 254 genes identified 15 genes (*Prkcb*, *Cysltr1*, *Flt1*, *Tnf*, *Tlr9*, *Tlr2*, *Il22ra2*, *Maff*, *Ccr2*, *Ccl8*, *Ptgfr*, *Cxcl1*, *Tslp*, *Alox12*, and *Ptger2*) that were differentially expressed to a significant extent in the skin at 6 h, but not at 24 h or 72 h, after lewisite exposure, and 14 genes (*Nos2*, *Ccr1*, *Chi3l3*, *Ccl17*, *H2-Eb1*, *Cxcr2*, *Cxcl5*, *Ptgs2*, *Oasl1*, *Ccl20*, *Tgfb2*, *Map2k6*, *Il23r*, and *Ccl22*) that were differentially expressed to a significant extent mainly at 24 h or 72 h after exposure (**Figures 3D, E**). The changes in the expression level of several of these genes correlated with the phases of SG assembly and disassembly after lewisite exposure: 9, 11, and 8 genes were significantly upregulated and 13, 8, and 6 genes were significantly downregulated, respectively, 6, 24, and 72 h after lewisite exposure (**Figure 3F**). Moreover, the expression of 4 genes, namely *Hmgb2*, *Il6*, *Csf3*, and *Ccl24*, was significantly altered at each time point. Among these genes, *Csf3* and *Il6* were significantly upregulated, while *Hmgb2* and *Ccl24*, which are involved, respectively, in nucleosome and cytoskeleton (29) assembly (among other functions), were significantly downregulated following lewisite exposure (**Figure 3G**). *Csf3* and *Il6* are also induced in murine skin following sulfur mustard exposure (30). Likewise, we found a correlation between the temporal pattern of SG assembly and disassembly and the temporal pattern of tissue damage in the lewisite-exposed skin sections of mice. TUNEL assays revealed the presence of only a few TUNEL-positive hair follicle keratinocytes 6 h after lewisite exposure, but widespread presence of TUNEL-positive cells 24 h after exposure, covering both inter-follicular and follicular epidermal keratinocytes was noted, and this effect was further enhanced 72 h after exposure (**Figure 3H**).

### The RBPs Roquin and Regnase-1 Underlie the Pathogenesis of Arsenical-Mediated Cutaneous Inflammation

**Figure 4:**
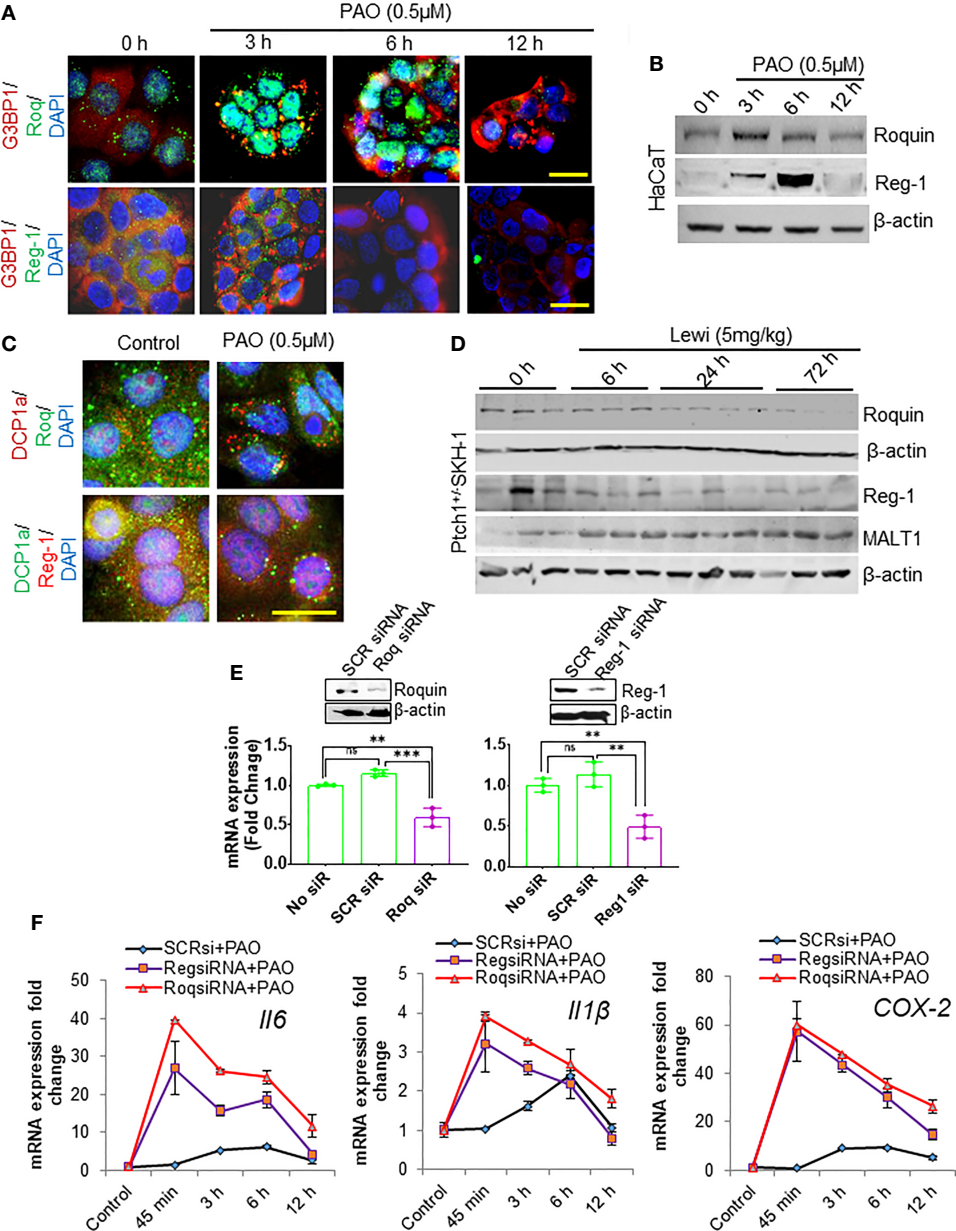
Roquin and Regnase-1 regulate the PAO-mediated inflammatory response during SG assembly and disassembly. **(A)** Representative immunofluorescence imaging of the time-dependent effects of PAO (0.5 µM) exposure on G3BP1 (red) and Roquin (green, top panel) or Regnase-1 (green, bottom panel) expression in HaCaT cells. Colocalization (yellow) of Roquin with G3BP1-positive SGs peaked 3 h after exposure (assembly phase) and decreased thereafter (disassembly phase), while Regnase-1 did not localize to SGs. Nuclei were stained with DAPI. (scale bar 50µm). **(B)** Representative Western blot showing the time-dependent effects of PAO (0.5 µM) exposure on Roquin and Regnase-1 expression in HaCaT cells. **(C)** Representative immunofluorescence imaging of the expression of the P body marker DCP1a (upper panel, red; lower panel, green) and Roquin (green, upper panel) or Regnase-1 (red, lower panel) in HaCaT cells 3 h after PAO (0.5 µM) exposure. Nuclei were stained with DAPI. (scale bar 50µm). **(D)** Representative Western blots showing the time-dependent effects of cutaneous lewisite (5 mg/kg) exposure on Regnase-1, Roquin, and MALT1 expression in the skin of Ptch1^+/-^/SKH-1 mice. β-actin was used as the endogenous loading control. **(E)** Representative Western blots (insets) and bar graphs showing, respectively, the effects of targeted siRNA-mediated knockdown on the protein and mRNA expression of Roquin (left) and Regnase-1 (right) in HaCaT cells. Scrambled siRNA was used as a negative control. **(F)** Quantitative analysis of the time-dependent effects of PAO (0.5 µM) exposure on *Il6*, *Il1β* and *COX-2* mRNA expression in HaCaT cells treated without or with Roquin- or Regnase-1-specific siRNA. SCR siRNA, scrambled siRNA; Roq, Roquin; Reg, Regnase. p < 0.01**, p < 0.001*** show significant level. ns, non significant.

The RBPs Roquin and Regnase-1 have critical roles in the degradation of inflammation-related mRNAs and in the maintenance of immune homeostasis (31). However, the role of these RBPs in the development of tissue injury, including the onset of inflammatory responses, after chemical exposure remains mostly undefined. In response to pathogen challenged cells Roquin is targeted to SGs, presumably to degrade translationally inactive mRNAs, particularly those that may translate to pro-inflammatory mediators (31). We confirmed by its co-localization with SG-forming G3BP1 that Roquin localizes to PAO-induced SGs in cultured HaCaT cells (**Figure 4A**). The expression of Roquin increased during the SG assembly phase (3 h after exposure) but diminished thereafter over the course of SG disassembly (6-12 h after exposure) (**Figures 4A, B**). In contrast, Regnase-1, a ribosome-localizing protein was largely absent from PAO-induced SGs (**Figure 4A**) as previously reported (31). Surprisingly, Regnase-1 expression was also apparent 3 h after PAO exposure. Unlike Roquin expression, however, the highest expression of Regnase-I was detected 6 h after exposure but diminished thereafter and was nearly absent 12 h after exposure (**Figure 4B**).

In response to stress, non-translating mRNAs are mainly dumped into processing bodies (P-bodies), which share some properties with SGs and participate in the degradation of mRNA (32). Therefore, we also examined the recruitment of Roquin and Regnase-1 to P-bodies. Immunofluorescence staining for DCP1a, a marker of P-bodies, revealed that PAO exposure also induced the formation of P-bodies (**Figure 4C**). However, neither Roquin nor Regnase-1 co-localized prominently with DCP1a (**Figure 4C**). Parallel to our *in vitro* data, Roquin and Regnase-1 expression in lewisite-treated murine skin decreased in a time-dependent manner (**Figure 4D**). However, in vehicle-treated control skin of Ptch1^+/-^/SKH-1 mice, we found detectable levels of these proteins. Given that the paracaspase mucosa-associated lymphoid tissue lymphoma translocation protein 1 (MALT-1) regulates the expression of these RNA-degrading proteins, we also determined the kinetics of MALT1 expression. MALT1 levels increased over time remained elevated 72 h after exposure (**Figure 4D**). To further confirm the role of Roquin and Regnase-1 in arsenical-mediated inflammation, we ablated the expression of each protein using RNAi approaches in HaCaT keratinocytes. Transfection with the respective target-specific siRNAs led to significant knockdown of Roquin and Regnase-1 expression at the mRNA and protein levels (**Figure 4E**). Ablation of either Roquin or Regnase-1 significantly augmented the PAO-induced increase in the expression of *Il6*, *Il1β*, and *COX-2* mRNA (**Figure 4F**). Interestingly, the PAO-mediated induction of these pro-inflammatory mediators also started much earlier, beginning as early as 45 min after exposure, in cells transfected with Roquin- or Regnase-1-siRNA.

### Alterations in SG Regulation Are Linked to Arsenical-Mediated Inflammation in Murine Skin

**Figure 5:**
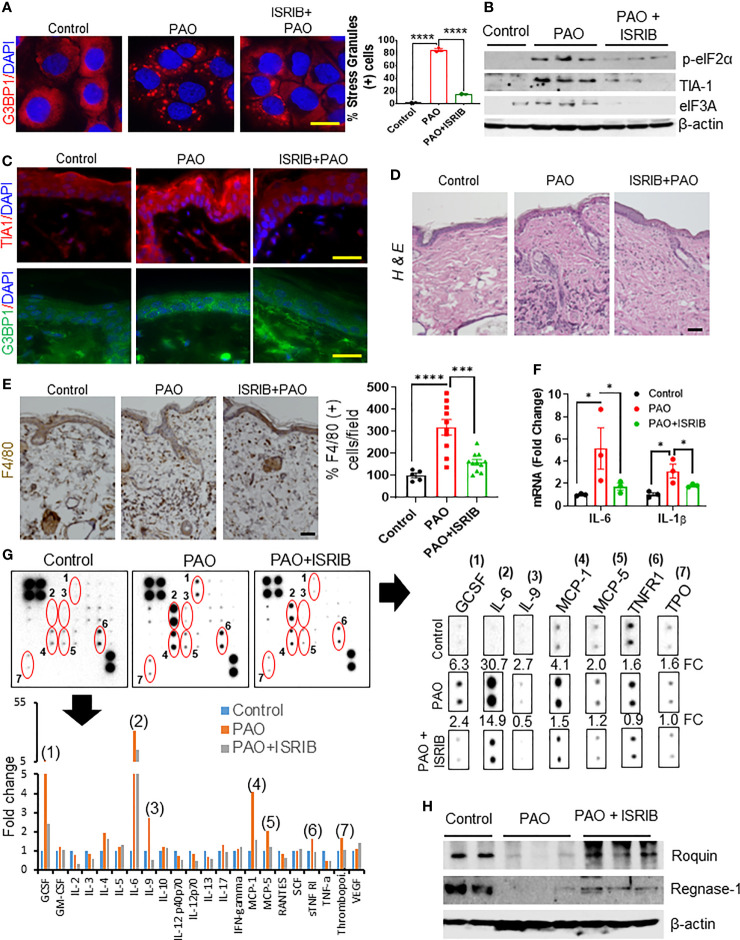
ISRIB treatment protects against arsenical-mediated inflammation by attenuating Roquin and Regnase-1 degradation in murine skin. **(A)** Left: representative immunofluorescence imaging of G3BP1-positive SGs (red) in HaCaT cells 3 h after co-treatment with ISRIB (100 nM) and PAO (0.5 µM). Nuclei were stained with DAPI. Right: Quantitative analysis of the percentage of cells expressing SGs in each treatment group (scale bar 50 µm). **(B–H)** Effects of topical ISRIB (200 µg/mouse) treatment 5-10 min after cutaneous PAO (100 µg/mouse) exposure in Ptch1^+/-^/SKH-1 mice. Mice were treated for 6 h before sacrifice. **(B)** Representative Western blot showing the expression of p-eIF2α, TIA1, and eIF3A after treatment in skin tissue lysates from each treatment group. β-actin was used as the endogenous loading control. **(C)** Representative immunofluorescence imaging of TIA1 (red) and G3BP1 (green) expression in skin sections from each treatment group. Nuclei were stained with DAPI (scale bar 50 µm). **(D)** Representative images of H&E-stained skin sections from each group showing the infiltration of inflammatory immune cells (scale bar 50 µm). **(E)** Left: representative images of skin sections from each treatment group showing the expression of the macrophage marker F4/80, detected by immunohistochemistry. Right: quantitative analysis of the percentage of F4/80 cells per field in each treatment group (scale bar 50 µm). **(F)** Quantitative analysis of the relative expression of *Il1β* and *Il6* mRNA, assessed by RT-PCR, in skin sections from each treatment group. **(G)** The relative expression of specific cytokines in skin tissue lysates was assessed by cytokine array. Skin lysates from 4–5 mice from each treatment group were pooled and incubated with the mouse cytokine antibody array membrane. Representative images of the cytokine arrays (top panel) and the histogram depicting densitometry data (bottom panel) are shown. The expression of GCSF (1), IL-6 (2), IL-9 (3), MCP-1 (4), MCP-5 (5), TNFR1 (6) and thrombopoietin (TPO) (7) were significantly higher in PAO-treated skin lysates than in controls but the increased expression was reduced more than 50% in the mice treated with ISRIB after PAO. Cropped array images of these cytokine mediators are presented separately (right panel). Densitometry data were analyzed by excel-based analysis software tools. FC (fold change) **(H)** Representative Western blots showing Roquin and Regnase-1 expression in skin lysates from each treatment group. β-actin was used as the endogenous loading control. p < 0.05*, p < 0.001***, p < 0.0001**** show significant level.

Previous reports have described SG dynamics in response to various stressors as both dependent and independent of eIF2α phosphorylation (33). However, we observed that lewisite- and PAO-induced phosphorylation of eIF2α is temporally associated with the dynamics of SG assembly and disassembly. Therefore, we investigated the potential role of p-eIF2α in the temporal regulation of arsenical-induced inflammatory responses, using the small molecule ISRIB. As also described earlier, ISRIB is a potent inhibitor of the integrated stress response (ISR) and acts by blocking the phosphorylation of eIF2α (34). Co-treatment with PAO and ISRIB (100 nM) substantially reduced the PAO-induced formation of SGs in keratinocytes (**Figure 5A**). Similarly, topical application of ISRIB (200 µg) on mouse skin 5-10 min after PAO treatment significantly reduced the phosphorylation of eIF2α and concomitantly reduced the expression of the SG-promoting proteins TIA1, and eIF3A otherwise observed 6 h after PAO exposure (**Figures 5B, C**).

H&E-staining of skin sections further showed that topical application of ISRIB after PAO challenge reduced the infiltration of inflammatory leukocytes in the epidermal/dermal compartments (**Figure 5D**). Similarly, IHC staining for the macrophage biomarker F4/80 showed that ISRIB treatment reduced the PAO-induced increase in this cell population (**Figure 5E**). RT-PCR analysis also showed significant downregulation of PAO-induced pro-inflammatory cytokines (*Il1β* and *Il6*) in the skin of mice treated with ISRIB after PAO exposure (**Figure 5F**). Furthermore, analysis of results from a mouse cytokine antibody array revealed significant induction of GCSF, IL-6, IL-9, MCP-1, MCP-5, TNFR1, and thrombopoietin (TPO) in skin samples after 6 h PAO exposure compared to control (**Figure 5G**). Remarkably, the expression of these inflammatory mediators was diminished significantly in mice treated with ISRIB after PAO exposure (**Figure 5G**). Finally, Western blot analysis of skin tissue lysates showed that ISRIB treatment significantly attenuated the PAO-mediated reduction in the expression of Roquin and Regnase-1 as well (**Figure 5H**). These results further demonstrate that arsenical-induced p-eIF2α-dependent SG formation underlies the pathogenesis of murine skin inflammation *via* regulation of Roquin and Regnase-1.

### SG Regulation Is Associated With Arsenical-Mediated Tissue Damage and Apoptosis in Murine Skin

**Figure 6:**
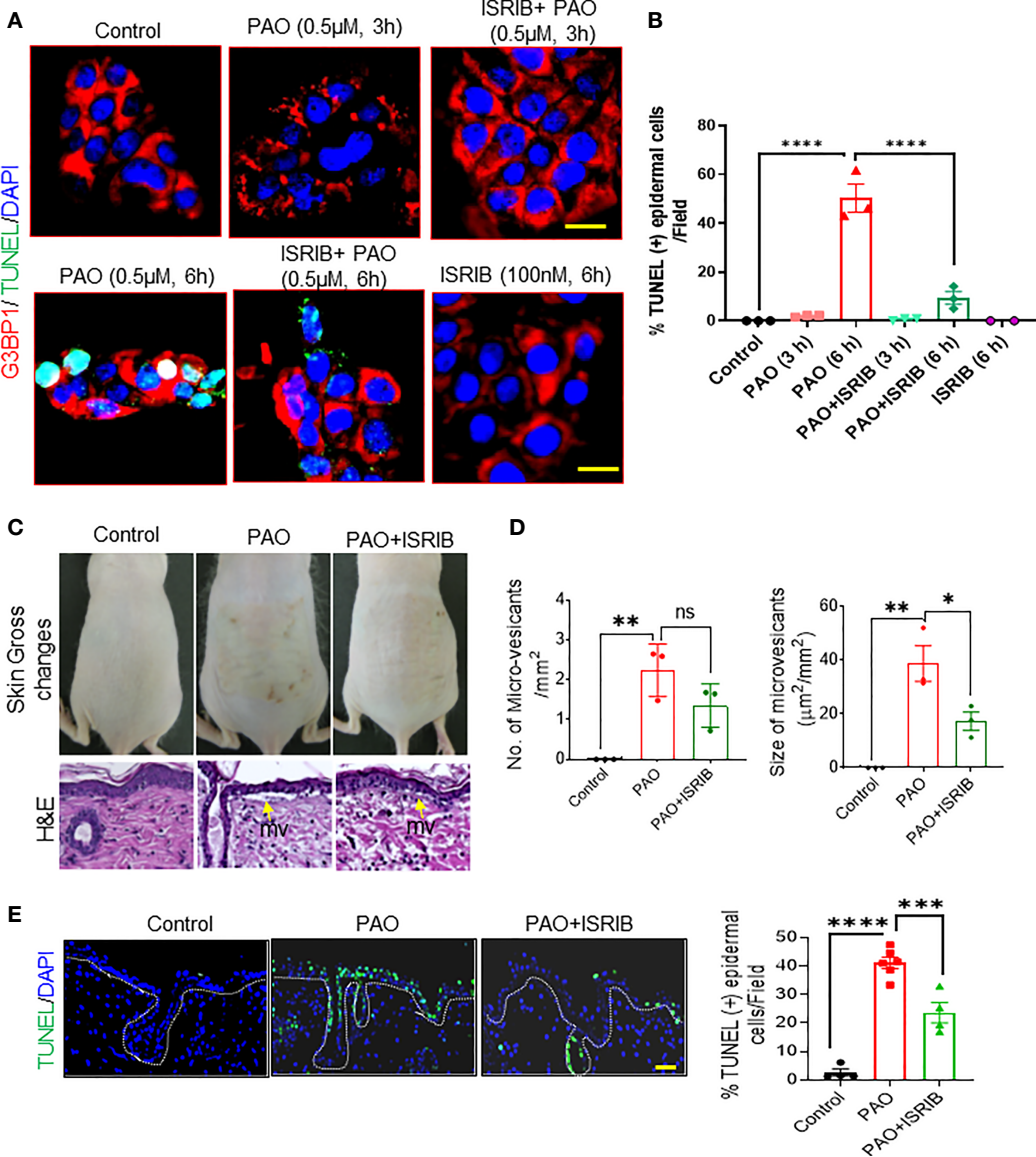
ISRIB protects PAO-mediated cell death and tissue injury in murine skin. **(A, B)** Effects of co-treating HaCaT cells with PAO (0.5 µM) and ISRIB (100 nM). **(A)** Representative immunofluorescence imaging of G3BP1 and TUNEL expression over time in each treatment group. Nuclei were stained with DAPI (scale bar, 50 µm). **(B)** Quantitative analysis of the changes in the percentage of TUNEL-positive cells over time in each treatment group. **(C–E)** Effects of topical ISRIB (200 µg/mouse) treatment 5-10 min after cutaneous PAO (100 µg/mouse) exposure in Ptch1^+/-^/SKH-1 mice. **(C)** Top panel: representative photographs showing the topical efficacy of ISRIB treatment in preventing the gross changes in skin normally observed 6 h after PAO exposure. Bottom panel: representative H&E-stained skin sections showing the histologic effects of each treatment. Yellow arrows point to microvesicants. **(D)** Quantitative analysis of the number (left histogram) and size (right histogram) of microvesicants detected in each treatment group. **(E)** Left: Representative immunofluorescence imaging of TUNEL-positive epidermal cells in skin sections from each treatment group. Nuclei were stained with DAPI (scale bar 50 µm). Right: Quantitative analysis of the percentage of TUNEL-positive cells in skin sections from each treatment group. Data are expressed as mean ± SEM. p < 0.05*, p < 0.01**, p < 0.001***, p < 0.0001**** show significant and ns- Non significant.

Next, we examined the effects of ISRIB on PAO-induced cell death in cultured human skin keratinocytes and in murine skin. Immunostaining in cultured keratinocytes revealed G3BP1-positive keratinocytes in PAO-induced SG assembly phase (at 3 h) were negative for TUNEL staining, (**Figures 6A, B**). However, by the beginning of the SG disassembly phase (6 h after exposure), in which cytoplasmic dispersal of SG-associated mRNA and proteins occurs, about 50% of keratinocytes were positive for TUNEL staining (**Figures 6A, B**). Co-treatment with ISRIB significantly diminished SG formation at 3 h and the percentage of TUNEL-positive cells at 6 h (**Figures 6A, B**).

*In vivo*, ISRIB topically administered 5-10 min after PAO remarkably decreased the skin damage caused by PAO, in which included cutaneous erythema, wrinkling, increased thickness (**Figure 6C**) and micro-vesicants (microblister) formation (**Figure 6D**). Specifically, ISRIB treatment reduced the number and size of PAO-induced micro-vesicants (**Figures 6C, D**). ISRIB also protected skin cells, including epidermal keratinocytes, against PAO-induced apoptosis (**Figure 6E**). These protective effects were more pronounced in cells of the interfollicular epidermis than in hair follicle–associated keratinocytes (**Figure 6E**), perhaps because hair absorbs arsenicals and thus provides a constant source of mild insult. However, it should be noted that in the hairless mice used here, the hair follicles are rudimentary and hair are fragile.

## Discussion

In this study, we provide strong evidence that SGs regulate the molecular pathogenesis and associated inflammatory response induced by cutaneous arsenical-exposure. Previously, we demonstrated that a single exposure to lewisite or PAO activates the unfolded protein response (UPR) signaling pathway, which is involved in the pathogenesis of cutaneous lesions (2, 12). Our data further demonstrated that unfolded proteins accumulated as a consequence of arsenical-induced ER stress, and if not folded properly in timely manner, the unfolded and misfolded proteins may ultimately become the cause of toxic manifestations of these highly hazardous chemicals. In the current study, we provide further evidence that accumulation of unfolded proteins leads to induction of SG formation and that the temporal regulation of SG assembly and disassembly correlates with the onset and progression of inflammation and tissue damage caused by arsenical exposure. Thus, this study has identified a key mechanism by which arsenicals incapacitate the defense response in the skin to manifest toxicity.

A number of molecular events are reported to be involved in the formation of SGs. One of the key events includes the phosphorylation of eIF2α, which in turn reduces the ternary complex, eIF2-GTP-tRNAiMet and that leads to initiation of SG assembly, at least by certain chemicals, as SG formation can also occur independent of eIF2α phosphorylation in other models of ER stress such as those induced by sorbitol (20, 33). We confirm here that arsenical-induced SG formation is associated with eIF2α phosphorylation, as treatment with ISRIB blocks the arsenical-induced phosphorylation of eIF2α and consequent SG formation (34). ISRIB treatment also significantly attenuated the inflammation and tissue injury induced in murine skin by topical application of arsenicals. SGs formed in response to stress contribute to cell survival not only by suppressing translation but also by sequestering some apoptosis regulatory signaling molecules. For example, the signaling proteins RACK1 (35), Raptor (36) and ROCK1 (37) were shown to be sequestered in SGs, which can affect apoptosis and promote cell survival. Our pathway analysis data also found that various proteins such as DDX5/6, RACK1, HSP90AA1, translation initiation factors, TIA1 and AGO1/2 from various canonical pathways as shown in **Figure 2C** are also sequestered in lewisite-induced SGs. This sequestration could determine the protein translation landscape and thereby may prevent the early onset of skin injury. SGs also contain many RBPs that regulate mRNA stability, structure, and function (38). Regnase-1 and Roquin are RBPs that degrade inflammation-related mRNAs and thus help maintain immune homeostasis (39, 40). This study revealed that both Roquin and Regnase-1 are important in regulating the arsenical-induced inflammatory response. Our observations that arsenical exposure significantly reduces the expression of these proteins, which in turn augments the production of proinflammatory cytokines, is consistent with the notion that dynamic regulation of both of these proteins is key to inducing robust inflammation following cutaneous exposure to arsenicals. Previously published data demonstrated that although Regnase-1 and Roquin regulate a common set of inflammation-related genes, each performs this function at distinct subcellular organelles. Regnase-1 specifically cleaves and degrades translationally active mRNAs at ribosomes, whereas Roquin controls translationally inactive or halted mRNA in the ER or at SGs (31). Our observations also demonstrate increased expression of Roquin and Regnase-1 early after arsenical exposure, but that only Roquin is targeted to SGs. However, after SG disassembly has begun, the expression of both Roquin and Regnase-1 decreases, suggesting the highly controlled and concerted regulation of each protein in the pathogenesis of cutaneous injury caused by arsenical exposure. The observation that siRNA-dependent ablation of both Regnase-1 and Roquin leads to substantial accumulation of inflammation-related mRNAs in keratinocytes treated with PAO further suggest the significant role of these RBPs in arsenical-induced injury. Indeed, RNAseq analyses demonstrated that in lewisite-treated skin, a panel of 4 genes (Mex3b, *Rch31*, *Eif4a1*, and *Ran*) is regulated by both Roquin and Regnase-1. In addition, we found that a panel of 4 genes (*Eif3b*, *Calr*, *Atxn2l*, and *Etf1*) is specifically regulated by Roquin while a panel of 7 different genes (*G3bp1*, *Ilf2*, *Cpeb2*, *Celf1*, *Rps3*, *Eif4h*, and *Rbm3*) is specifically regulated by Regnase-1 (**Supplementary Figure S2**).

**Figure 7:**
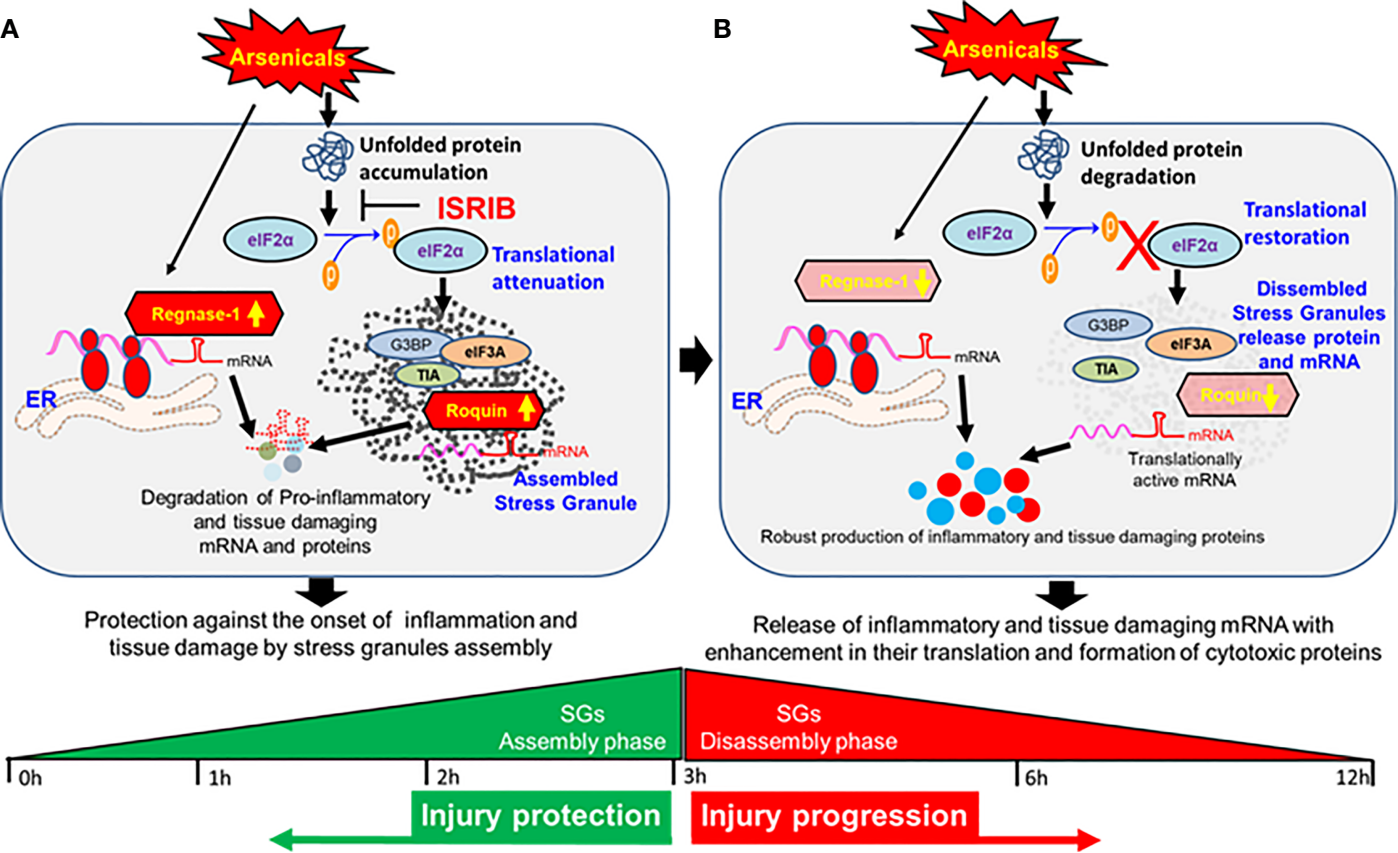
Dynamics of stress granule and global translation attenuation in the pathogenesis of vesicant arsenical toxicity. **(A)** Flow diagram depicting protection against the onset of inflammation and tissue damage at early hours by stress granules in assembly phase. **(B)** Flow diagram showing release of inflammatory and tissue damaging mRNAs with enhancement in their translation and formation of cytotoxic proteins at late hours when stress granules disassemble.

The finding that treatment with ISRIB soon after PAO exposure prevents the loss of Roquin and Regnase-1 expression in murine skin and thereby blocks the phosphorylation of eIF2α suggests a link between the attenuation of ISR and induction of RBPs that, while restoring tissue homeostasis of target inflammatory signaling. These studies are highly novel in demonstrating the role of the RBPs in cutaneous chemical injury. However, further study is needed to elucidate the mechanism by which these RBPs are regulated in chemical-induced inflammation and tissue disruption. The flow diagram shown as **Figure 7** summarizes these mechanistic effects of arsenicals at assembly and disassembly phases of SGs (**Figure 7**).

The only known antidote for arsenicals is British Anti-Lewi (BAL), which is a chelating agent and acts by forming inorganic complex with arsenic (27). Importantly, however, treatment with BAL is not very effective, is also extremely painful, and manifests toxic side effects (41, 42). Our finding that ISRIB is likely effective in attenuating arsenical injury suggests this small molecule as a novel mechanism-based medical counter measure (MCM) therapeutic for vesicant tissue injury. Our observations confirming previous results that ISRIB is well-tolerated, particularly, following acute administration (43) is consistent with the notion that it could be developed as an effective and safe MCM for lewisite and other arsenicals.

## Conclusions

In summary, we have documented the critical role of temporal regulation of SGs in regulating skin defense mechanism against exposure to warfare vesicant chemicals. We have also revealed the mechanistic underpinning of the robust inflammatory signaling associated with cutaneous blistering by these vesicants which is mediated by the dysregulation of proinflammatory mRNA degrading RNA binding proteins regnase and roquin. Finally, we identified a small molecule ISRIB that may be developed into a highly effective and clinically relevant mechanism-based antidote to protect against the debilitating effects of cutaneous arsenical exposure.

## Data Availability Statement

All data relevant to this study are included in this article and in supplementary materials. The in-house transcriptome dataset can be acquired through the reasonable request to the corresponding author. However, publicly available data sets which were analyzed in this study can be found here: http://proteomecentral.proteomexchange.org/cgi/GetDataset?ID=PXD010520.

## Ethics Statement

The animal study was reviewed and approved by the Institutional Animal Care and Use Committee (IACUC) of the University of Alabama at Birmingham and MRIGlobal.

## Author Contributions

RS, SM, and BM conducted experiments and collected data. RS analyzed *in vitro* and *in vivo* data. BM and MM performed bioinformatics analyses. RS and MA designed the study and wrote the manuscript. MG, AA, MM and MA contributed to the study idea and editing. RS, BM, MG, AA, MM, and MA finalized the manuscript for submission.

## Funding

This work was supported by the Countermeasures Against Chemical Threats (Counteract) Program, NIH grants number U01NS095678, U01AR078544 and U54ES030246 to MA. The study sponsors had no involvement in the study design, collection, analysis and interpretation of data, the writing of the manuscript or the decision to publish the manuscript.

## Conflict of Interest

The authors declare that the research was conducted in the absence of any commercial or financial relationships that could be construed as a potential conflict of interest.

## Publisher’s Note

All claims expressed in this article are solely those of the authors and do not necessarily represent those of their affiliated organizations, or those of the publisher, the editors and the reviewers. Any product that may be evaluated in this article, or claim that may be made by its manufacturer, is not guaranteed or endorsed by the publisher.
