# Supplementary material for: Dynamic Regulation of the Nexus Between Stress Granules, Roquin, and Regnase-1 Underlies the Molecular Pathogenesis of Warfare Vesicants
Source: Front Immunol. 2022 Jan 10;12:809365. doi: 10.3389/fimmu.2021.809365 (PMC8784689; doi:10.3389/fimmu.2021.809365)
Supplement: Supplementary file 1 [file Presentation_1.pptx]

## Slide 1
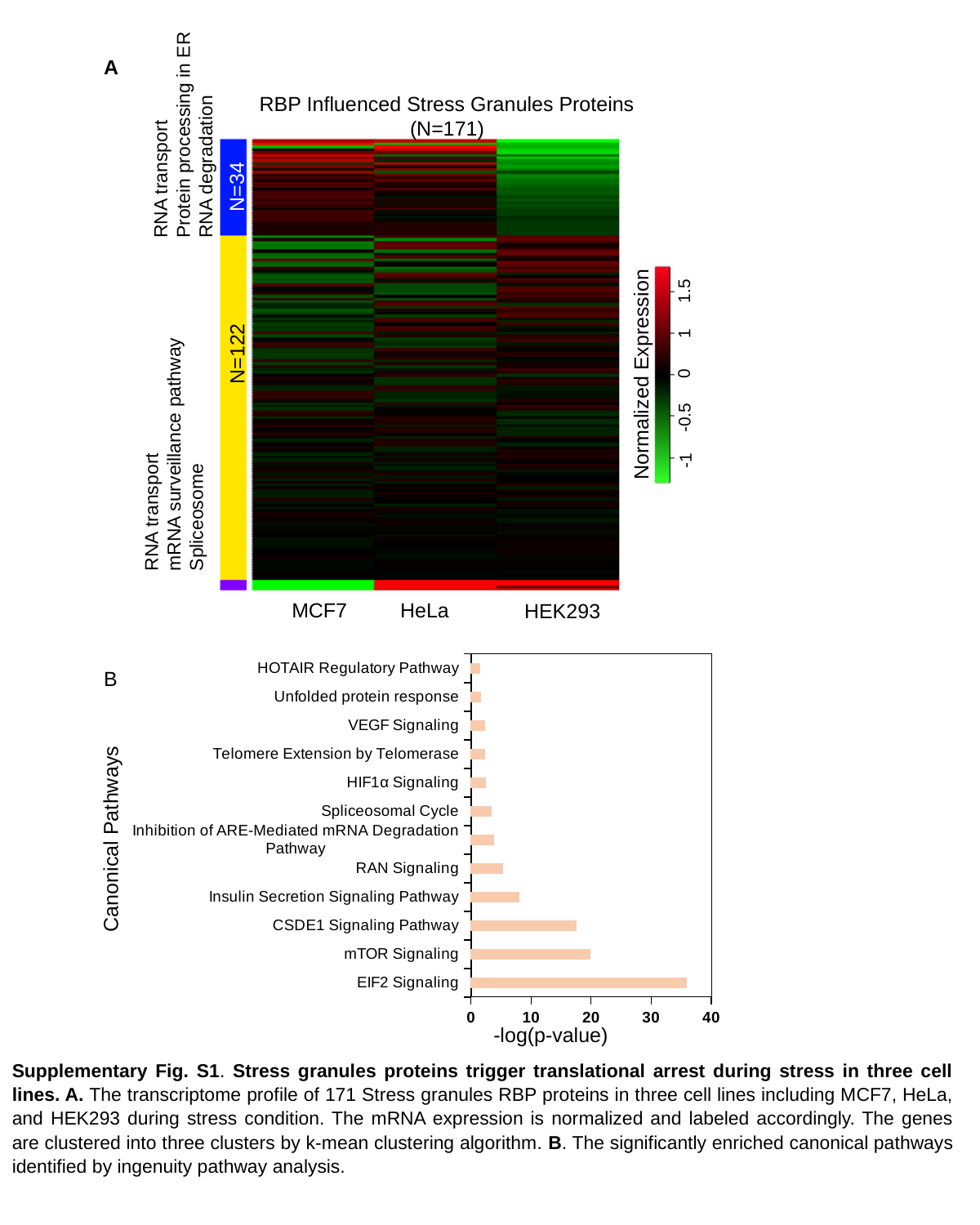

RNA transport
Protein processing in ER
RNA degradation
N=34
N=122
RNA transport
mRNA surveillance pathway
Spliceosome
MCF7
HeLa
HEK293
A
RBP Influenced Stress Granules Proteins (N=171)
Normalized Expression
0
1.5
1
-0.5
-1
### Chart
| Category | -log(p-value) |
|---|---|
| EIF2 Signaling | 35.9 |
| mTOR Signaling | 19.8 |
| CSDE1 Signaling Pathway | 17.5 |
| Insulin Secretion Signaling Pathway | 7.98 |
| RAN Signaling | 5.23 |
| Inhibition of ARE-Mediated mRNA Degradation Pathway | 3.75 |
| Spliceosomal Cycle | 3.36 |
| HIF1α Signaling | 2.38 |
| Telomere Extension by Telomerase | 2.29 |
| VEGF Signaling | 2.23 |
| Unfolded protein response | 1.56 |
| HOTAIR Regulatory Pathway | 1.51 |Canonical Pathways
-log(p-value)
B
Supplementary Fig. S1. Stress granules proteins trigger translational arrest during stress in three cell lines. A. The transcriptome profile of 171 Stress granules RBP proteins in three cell lines including MCF7, HeLa, and HEK293 during stress condition. The mRNA expression is normalized and labeled accordingly. The genes are clustered into three clusters by k-mean clustering algorithm. B. The significantly enriched canonical pathways identified by ingenuity pathway analysis.

## Slide 2
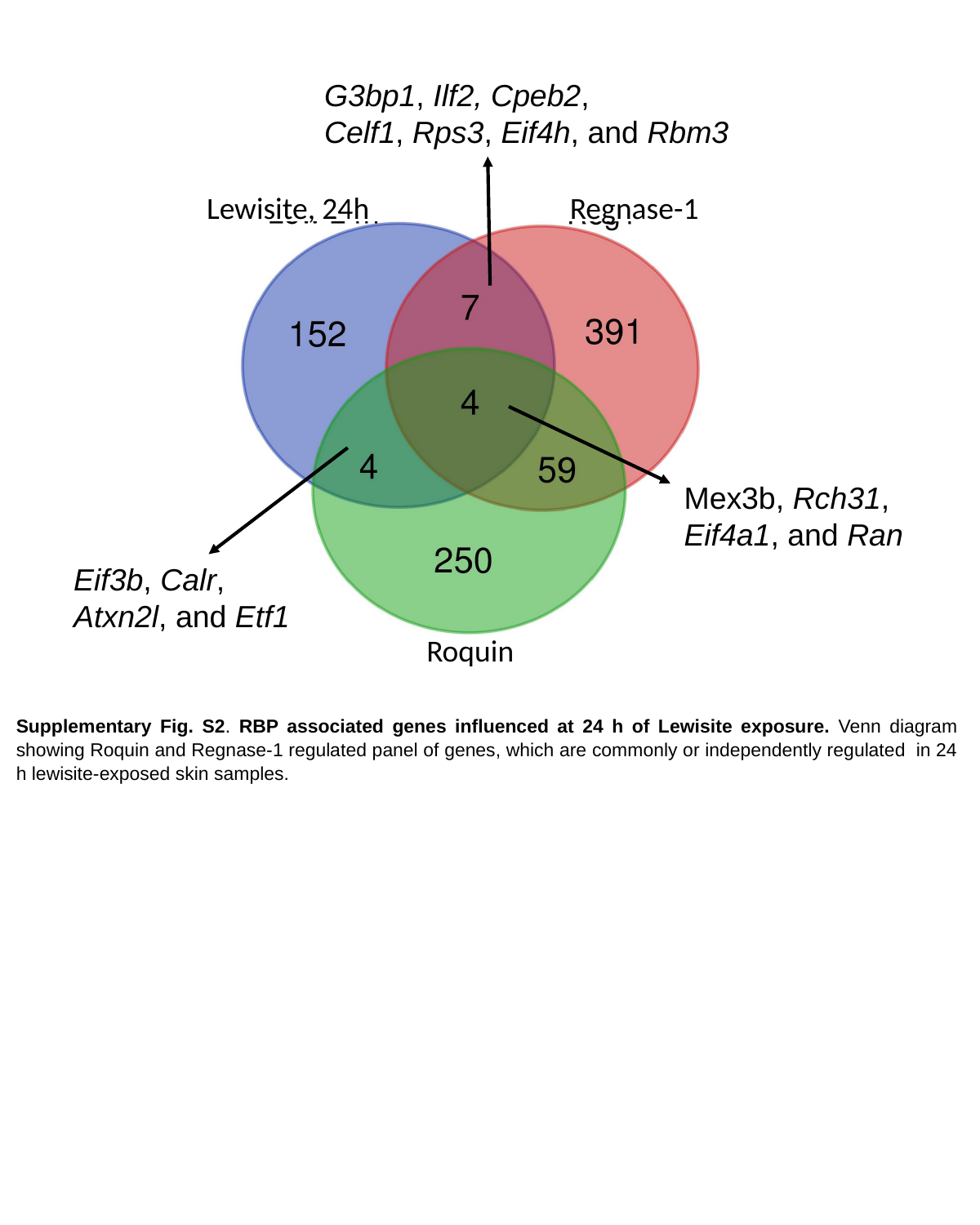

G3bp1, Ilf2, Cpeb2,
Celf1, Rps3, Eif4h, and Rbm3
Lewisite, 24h
Regnase-1
Mex3b, Rch31,
Eif4a1, and Ran
Eif3b, Calr,
Atxn2l, and Etf1
Roquin
Supplementary Fig. S2. RBP associated genes influenced at 24 h of Lewisite exposure. Venn diagram showing Roquin and Regnase-1 regulated panel of genes, which are commonly or independently regulated in 24 h lewisite-exposed skin samples.

## Slide 3
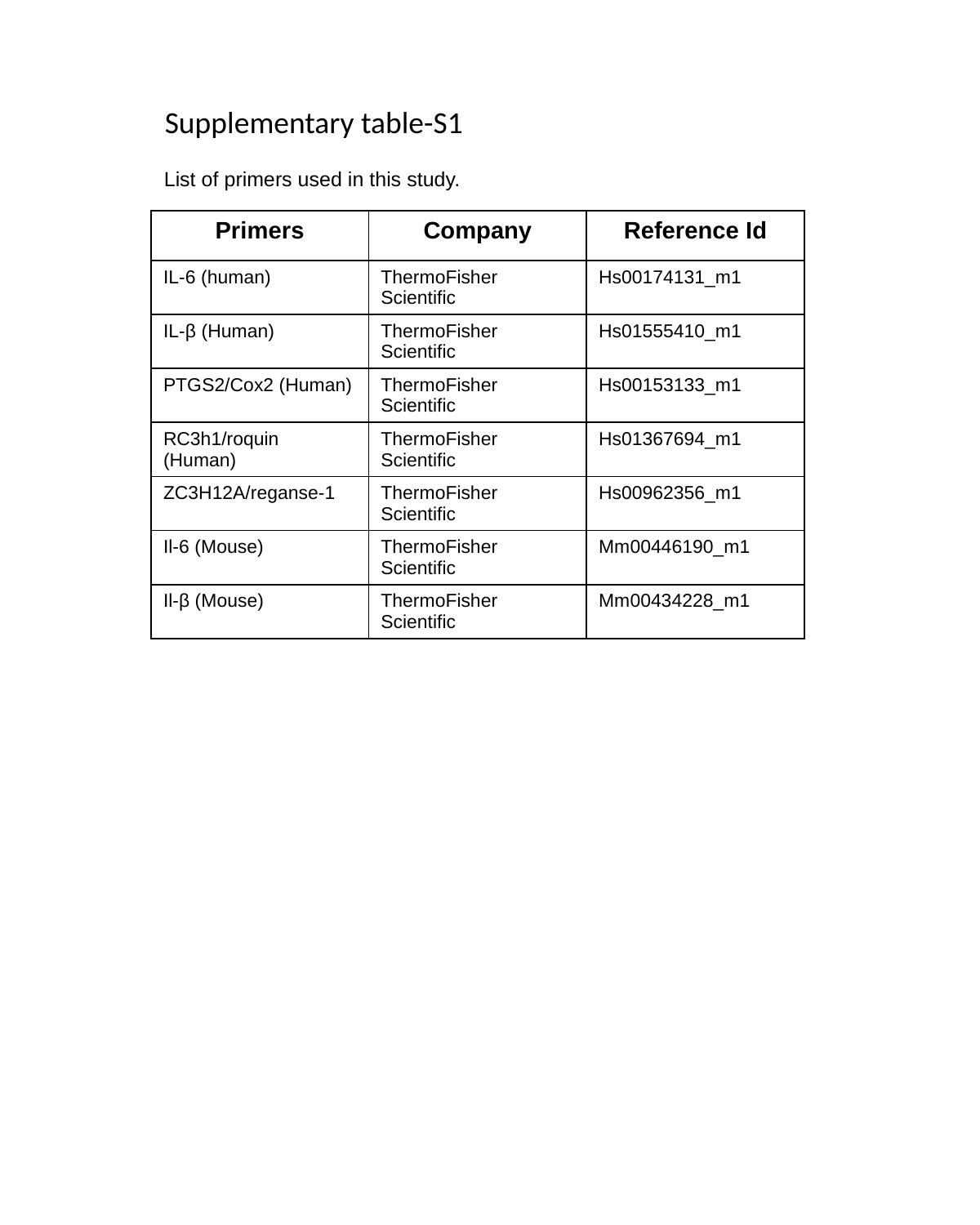

Supplementary table-S1
List of primers used in this study.
| Primers | Company | Reference Id |
| --- | --- | --- |
| IL-6 (human) | ThermoFisher Scientific | Hs00174131\_m1 |
| IL-β (Human) | ThermoFisher Scientific | Hs01555410\_m1 |
| PTGS2/Cox2 (Human) | ThermoFisher Scientific | Hs00153133\_m1 |
| RC3h1/roquin (Human) | ThermoFisher Scientific | Hs01367694\_m1 |
| ZC3H12A/reganse-1 | ThermoFisher Scientific | Hs00962356\_m1 |
| Il-6 (Mouse) | ThermoFisher Scientific | Mm00446190\_m1 |
| Il-β (Mouse) | ThermoFisher Scientific | Mm00434228\_m1 |

## Slide 4
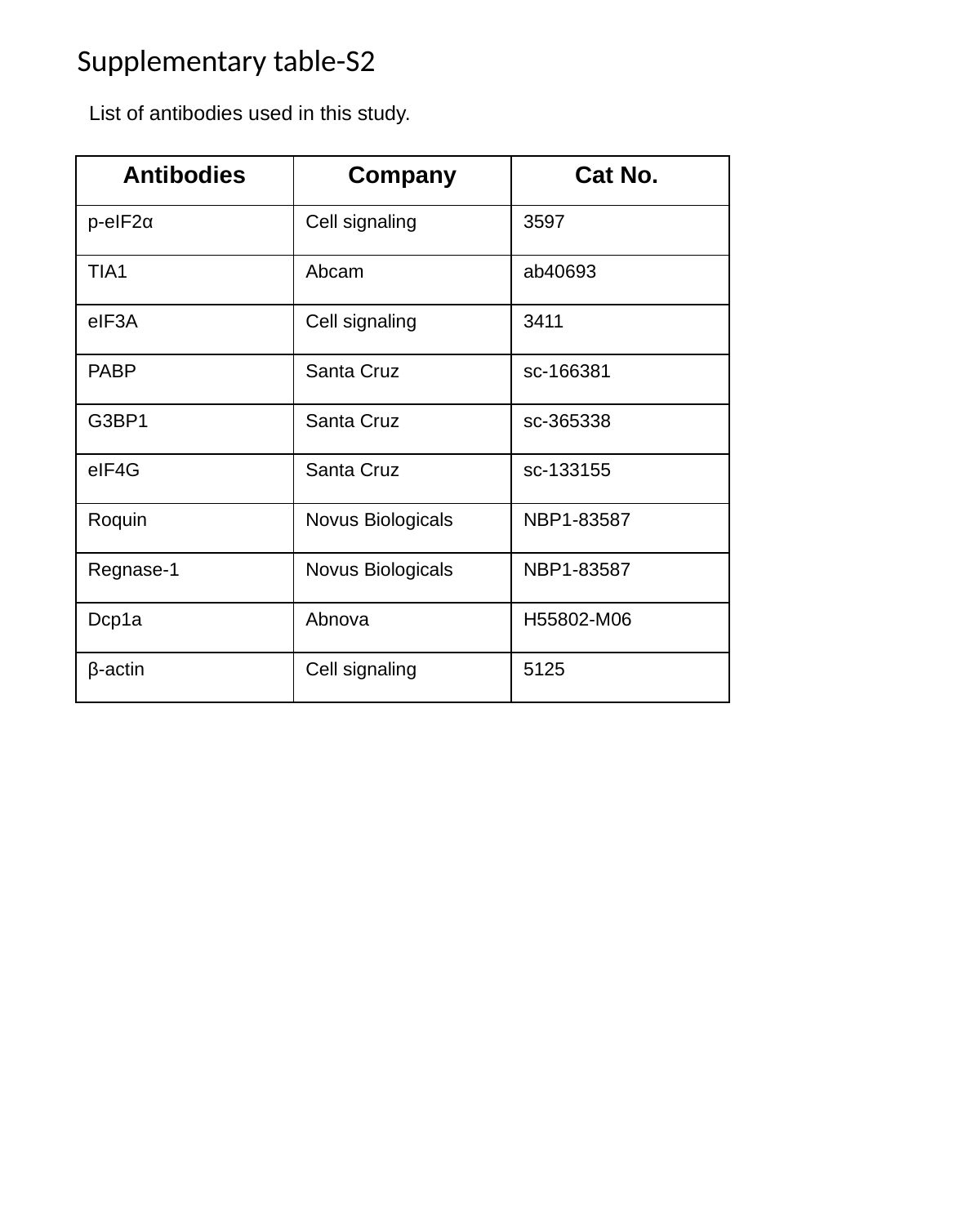

Supplementary table-S2
List of antibodies used in this study.
| Antibodies | Company | Cat No. |
| --- | --- | --- |
| p-eIF2α | Cell signaling | 3597 |
| TIA1 | Abcam | ab40693 |
| eIF3A | Cell signaling | 3411 |
| PABP | Santa Cruz | sc-166381 |
| G3BP1 | Santa Cruz | sc-365338 |
| eIF4G | Santa Cruz | sc-133155 |
| Roquin | Novus Biologicals | NBP1-83587 |
| Regnase-1 | Novus Biologicals | NBP1-83587 |
| Dcp1a | Abnova | H55802-M06 |
| β-actin | Cell signaling | 5125 |
